# A population based validation study of self-reported pensions and benefits: the Nord-Trøndelag health study (HUNT)

**DOI:** 10.1186/1756-0500-6-27

**Published:** 2013-01-23

**Authors:** Solbjørg Makalani Myrtveit, Anja M S Ariansen, Ingvard Wilhelmsen, Steinar Krokstad, Arnstein Mykletun

**Affiliations:** 1 Institute of medicine, University of Bergen, Bergen, Norway; 2 Norwegian Institute of Public Health, Division of Mental Health, Department of Public Mental Health, Bergen, Norway; 3 Department of Health Promotion and Development, Faculty of Psychology, University of Bergen, Bergen, Norway; 4 Faculty of social sciences, University of Bergen, Bergen, Norway; 5 Department of Medicine, Haraldsplass Deaconal Hospital, Bergen, Norway; 6 HUNT Research Centre, Department of Public Health and General Practice, Norwegian University of Science and Technology, Trondheim, Norway; 7 University of New South Wales, School of Psychiatry, Sydney, Australia

**Keywords:** Validity, Bias, Epidemiology, Non-responders

## Abstract

**Background:**

Measures of disability pensions, sickness certification and long-term health related benefits are often self-reported in epidemiological studies. Few studies have examined these measures, and the validity is yet to be established.

We aimed to estimate the validity of self-reported disability pension, rehabilitation benefit and retirement pension and to explore the benefit status and basic characteristics of those not responding to these items.

A large health survey (HUNT2) containing self-reported questionnaire data on sickness benefits and pensions was linked to a national registry of pensions and benefits, used as “gold standard” for the analysis. We investigated two main sources of bias in self-reported data; misclassification - due to participants answering questions incorrectly, and systematic missing/selection bias - when participants do not respond to the questions.

Sensitivity, specificity, positive (PPV) and negative (NPV) predicative value, agreement and Cohen’s Kappa were calculated for each benefit. Co-variables were compared between non-responders and responders.

**Results:**

In the study-population of 40,633, 9.2% reported receiving disability pension, 1.4% rehabilitation benefits and 6.1% retirement pension. According to the registry, the corresponding numbers were 9.0%, 1.7% and 5.4%. Excluding non-responders, specificity, NPV and agreement were above 98% for all benefits. Sensitivity and PPV were lower. When including non-responders as non-receivers, specificity got higher, sensitivity dropped while the other measures changed less.

Between 17.7% and 24.1% did not answer the questions on benefits. Non-responders were older and more likely to be female. They reported more anxiety, more depression, a higher number of somatic diagnoses, less physical activity and lower consumption of alcohol (p < 0.001 for all variables). For disability pension and retirement pension, non-responders were less likely to receive benefits than responders (p < 0.001). For each benefit 2.1% or less of non-responders were receivers. False positive responses were more prevalent than false negative responses.

**Conclusions:**

The validity of self-reported data on disability pension, rehabilitation benefits and retirement pension is high – it seems that participants’ responses can be trusted. Compared to responders, non-responders are less likely to be receivers. If necessary, power and validity can be kept high by imputing non-responders as non-receivers.

## Background

In epidemiological studies, self-reported information on disability pensions, sickness certification and long-term health related benefits is often used as exposure and/or outcome measures. The validity of such information can be challenged by different sources of biases [1,2]. For instance, individuals might choose not to participate, potentially leading to selection bias. Others might participate but not answer specific items. Participants might also answer questions incorrectly, leading to misclassification. Further, imputation procedures might introduce selection bias.

Multiple factors like sample population, cognitive abilities, recall time frame, questionnaire design, question comprehension and interpretation can affect the accuracy of self-reported information [3-7]. In general, research participants want to respond in ways that make them look as good as possible. For instance, in organizational behavioral research social desirability has been found significantly correlated with several widely used constructs like self-report of job performance, citizenship behavior and vitality [8,9]. Also other types of self-reported information seem affected by social desirability; for instance, a large downward bias in reporting food intake related to social desirability score has been found [10].

Reluctance to answer correctly or to answer at all can be seen when participants are questioned on issues perceived as stigmatizing or sensitive. Higher privacy provided in the information gathering process produce higher reported rates of alcohol consumption and other drug use [5]. Computer-assisted self-administered interviewing compared to computer-assisted personal interviewing result in higher reporting of both drug use and number of sexual partners [6]. However, participation rate seemed less affected by mode of data collection [6]. Also the validity of self-evaluated ability was associated with whether or not anonymity was guaranteed [7]. High intelligence, high achievement status and internal locus of control seem associated with more accurate evaluation of ability [7].

Whereas there are several studies of the validity of self-reported sickness-absence [11-21], there are only a few studies exploring validity of self-reported disability pension [15,21,22]. We are aware of no study investigating the validity of retirement pension or rehabilitation benefits. The studies that have been conducted mainly focused on the validity of answers, not investigating non-responders.

The aim of the current study was twofold: 1) to investigate the validity of self-reported disability pension, rehabilitation benefit and retirement pension, and 2) to explore the benefit status of participants not answering questions on benefits and pensions, and to explore demographic and health characteristics of non-responders. In order to do this, self-reported information from the population based dataset (HUNT2) was compared to a national register covering the total population.

## Methods

### Study population

Data from “Helseundersøkelsen i Nord-Trøndelag (the HUNT Study, HUNT2, 1995–1997)” were used. Nord-Trøndelag is one of 19 counties in Norway, and largely characteristic of the national population, though slightly less urban and with lower education attainment [23]. The HUNT2 Survey was conducted between August 1995 and March 1997. All inhabitants in Nord-Trøndelag County aged >19 (n = 93,898) years received mailed questionnaires and an invitation to a clinical examination. The participation rate was 69.5% (65.4% for men and 73.5% for women).

Most participants aged 70 years or older received a specialized questionnaire not including questions on benefit status and were therefor excluded from this study (N = 7,733). In the end, n = 40,633 (n = 18,979 men and n = 21,654 women), mean age 44.6 years (SD: 13.4 years, range: 19 to 87 years) completed the questionnaire containing information needed in this study, and constituted our study population.

Like in previous studies [24], the data from HUNT2 were linked to FD-trygd using a unique 11-digit identification number assigned to all individuals living in Norway. FD-trygd is a historical event database often used in epidemiological research in Norway [25,26]. It contains information on topics like demography, social conditions, social security, employment, search for work and income for the entire Norwegian population from 1992 an onwards.

By linking these two datasets, we were able to validate participants’ self-reported information on pensions/benefits (aim one), and to investigate non-responders and the possibility of systematic selection bias (aim two).

### Analytic samples

Investigations of validity were conducted using the entire study-population of n = 40,633. For exploration of characteristics of individuals not responding to the questions on benefits, information on co-variables was needed. Therefore, only individuals answering questions on co-variables were included and a subsample n = 34,262 was used for these analysis.

### Register based accurate information

Data on disability pension, rehabilitation benefits and retirement pension was found in FD-trygd, providing the base for evaluating self-reported information.

### Self-reported information on benefits

Participants in HUNT2 were asked if they at time of participation received any social security benefit or pension, with the tick-off-possibilities: “Disability pension”, “Retirement pension” or “Rehabilitation benefit”.

Disability pension is to ensure a subsistence income for individuals with wage earning capacity permanently impaired due to an illness or injury [27]. It can be granted a 100% or graded (if graded, almost always as 50% or higher) [28].

Rehabilitation benefits are usually granted individuals with at least 50% disability. The benefit is supposed to help the disabled individual back into a position where he or she is able to work and function in society. Even when individuals are less than a 100% disabled, and do have work ability left, most of the individual’s time, energy and abilities are required for successful rehabilitation. Thus, the individual cannot work and rehabilitation benefit is usually granted 100% [28].

Retirement pensions from the Norwegian National Insurance Scheme ensure all citizens an income in their old age. The benefit can be received in 20, 40, 50, 60, 80 or 100% from 62 years of age, but it is usually received as 100% from 67 years of age [29].

### Self-reported information on co-variables

Questions on co-variables were only asked for in HUNT2, with no corresponding objective information in FD-trygd. This data is therefore used as survey-data, and is not validated.

Gender was analyzed as a co-variable, as was marital status, grouped into “not married”, “married”, “separated/divorced” or “widow/widower”.

Participants’ health was evaluated using questions on somatic diagnoses and symptoms of common mental disorders. Somatic diagnoses were recorded by ticking off for present or past cardiac infarction, angina pectoris, stroke, asthma, diabetes and/or multiple sclerosis.

Anxiety and depression were measured using the “Hospital Anxiety and Depression Scale” (HADS) [30], a widely used self-report questionnaire [31-33]. In accordance with previous studies, a valid rating of depression and anxiety was defined as at least 5 completed items at each sub-scale (HADS-S and HADS-D) [33,34], and the recommended cut-off score of ≥8 was used in the descriptive table [31-33].

Health-related behavior was evaluated in line with previous studies [35,36]. The participants were asked “Do you smoke cigarettes, cigars and/or pipe daily” and grouped as smokers or non-smokers. Physical activity was evaluated by asking how often and for how long the participants engaged in both light and intense leisure-time physical activity. Amount of alcohol consumption was assessed using two questions: “Do you abstain from alcohol?” and “What is your normal consumption of alcoholic beverages within 14 days?”. Based on this, using a cut-off value of 15 units, participants were grouped to have “no consumption”, “moderate consumption”, or “high consumption”.

Questions on co-variables were answered by n = 34,262 individuals. This subsample was used for investigating characteristics of those not responding to the questions on benefits.

### Statistics

Aim one: Each benefit was evaluated separately using data sets from FD-trygd linked to HUNT2. The registered benefits in FD-trygd were set as the gold standard. For each benefit, a 2x3-table for comparison was made, displaying gold standard case and non-case against self-reported case, non-case and missing (Table 1). From these tables analyses were conducted. Excluding non-responders, sensitivity (% of true cases reported as cases), specificity (% of true non-cases reported as non-cases), positive predicative value (PPV) (% of those set as cases that are true cases), and negative predicative value (% of those set as non-cases that are true non-cases), agreement and Cohens Kappa were calculated for valid responses. Subsequently, the same measures were calculated again, with non-responders set as non-receivers.

**Table 1:** Self-reported versus public registry information on disability pension, rehabilitation benefits and retirement pension, the HUNT Study (HUNT2, 1995–1997), N = 40,633 aged 19–87 years

		**Registry based information (gold standard)**		
		**Recipient of pension**	**Non-recipient**	**Total**
Self-reported information	Disability pension	3,487 (93.3%)	251 (6.7%)	3,738 (100%)
	No disability pension	46 (0.2%)	29,675 (99.8%)	29,721 (100%)
	Missing response	129 (1.8%)	7,045 (98.2%)	7,174 (100%)
	Total	3,662 (9.0%)	36,971 (91.0%)	40,633 (100%)
	Rehabilitation benefits	419 (72.2%)	161(27.8%)	580 (100%)
	No rehabilitation benefit	94 (0.3%)	30,148 (99.7%)	30,242 (100%)
	Missing response	181 (1.8%)	9,630 (98.2%)	9,811 (100%)
	Total	694 (1.7%)	39,939 (98.3%)	40,633 (100%)
	Retirement pension	2,047 (83.2%)	414 (16.8%)	2,461 (100%)
	No retirement pension	39 (0.1%)	29,762 (99.9%)	29,801 (100%)
	Missing response	93 (1.1%)	8,278 (98.9%)	8,371 (100%)
	Total	2,179 (5.4%)	38,454 (94.6%)	40,633 (100%)

Cross-table: Self-reported information on benefits versus public registry information. Data provided in numbers and percentages. Percentages calculated to equal 100% in rows.

Aim two: A table comparing co-variables between those answering and those not answering the questions on benefits also made, using the subsample n = 34,262 (excluding those not answering questions on covariables). Chi-square test and two-tailed T-test were used to test if differences were significant.

STATA/IC 11 for Windows 7, PC, was used for all analyses.

### Ethics

The data used are not openly available but were approved for use by Regional Committees for Medical and Health Research Ethics of Mid-Norway, Norway. All the participants in HUNT2 gave their written consent upon inclusion.

## Results

### Study population

The study population consisted of n = 40,633 individuals. Among these, 53.3% were female and 3.3% were benefit receivers. The mean age 44.6 years (SD: 13.5 years, range: 19 to 87 years). The sample was predominantly Caucasian. Basic characteristics for the subsample answering questions on co-variables are presented in Table 2.

**Table 2:** Basic characteristics of study population with valid answers for co-variables, the HUNT Study (HUNT2, 1995–1997), N = 34,262 aged 19–87 years

	**Female (N = 17,808)**	**Male (N = 16,454)**	P-values*	**Total (N = 34,262)**
Female	17,808 (100%)	0 (0%)		17,808 (52.0%)
Age (mean (SD))	42.5 (12.9)	44.4 (13.0)	<.001	43.4 (13.0)
Anxiety	3,135 (17.6%)	2,083 (12.7%)	<.001	5,218 (15.2%)
Depression	1,544 (8.7%)	1,585 (9.6%)	.002	3,129 (9.1%)
Somatic diagnosis >0	1,878 (10.6%)	2,310 (14.0%)	<.001	4,188 (12.2%)
Physical activity			<.001	
*No*	3,720 (20.9%)	3,947 (24.0%)		7,667 (22.4%)
*Moderate*	9,497 (53.3%)	6,661 (40.5%)		16,158 (47.2%)
*Heavy*	4,591 (25.8%)	5,846 (35.5%)		10,437 (30.5%)
Consumption of alcohol			<.001	
*No consumption*	5,877 (33.0%)	3,176 (19.3%)		9,053 (26.4%)
*Moderate consumption*	11,788 (66.2%)	12,477 (75.8%)		24,265 (70.8%)
*Heavy consumption*	143 (0.8%)	801 (4.9%)		944 (2.8%)
Smoking	6,110 (34.3%)	4,973 (30.2%)	<.001	11,083 (32.4%)
Marital status			<.001	
*Married*	11,059 (62.1%)	10,078 (61.3%)		21,137 (61.7%)
*Not married*	4,585 (25.8%)	5,076 (30.9%)		9,661 (28.2%)
*Separated or divorced*	1,475 (8.3%)	1,109 (6.7%)		2,584 (7.6%)
*Widow/Widower*	688 (3.9%)	189 (1.2%)		877 (2.6%)
Self-reporting disability pension	1,529 (8.6%)	1,108 (6.7%)	<.001	2,637 (7.7%)
Self-reporting rehabilitation benefit	268 (1.5%)	226 (1.4%)	.087	494 (1.4%)
Self-reporting retirement pension	667 (3.8%)	906 (5.5%)	<.001	1,573 (4.6%)

*testing if there is a significant difference between genders, chi-square test/T-test.

### Measures of validity, aim one

**Figure 1:**
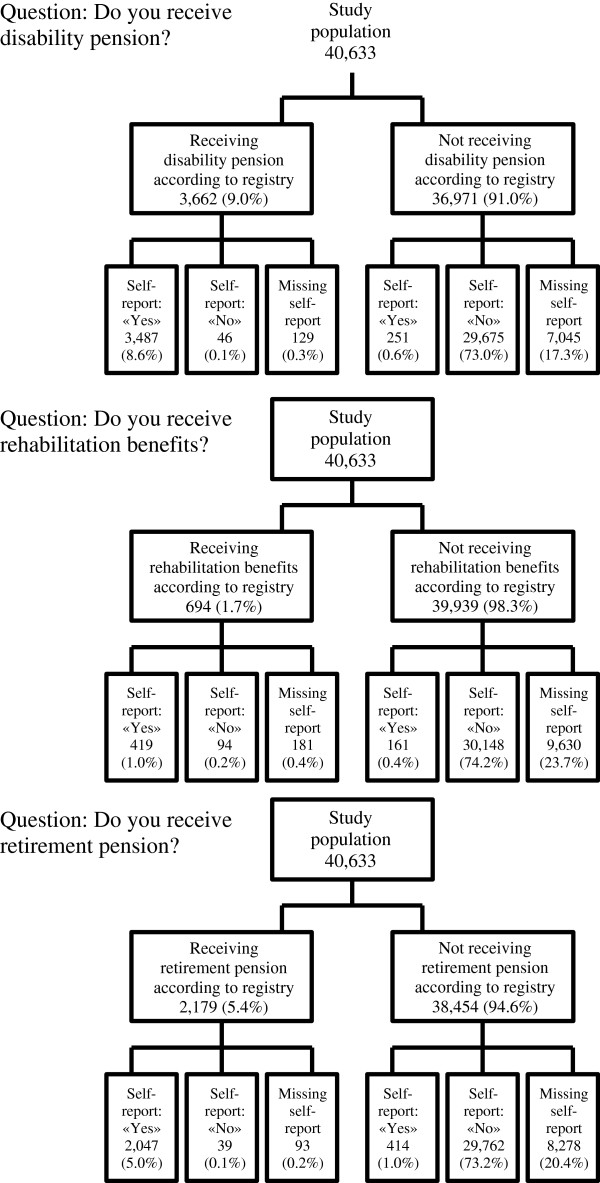
**Self-reported information versus registry information on disability pension, rehabilitation benefits and age retirement pension.** The HUNT Study (HUNT2, 1995-97), Norway.

For disability pension and retirement pension, the number of individuals reporting to be on the benefit was higher than the actual number of receivers according to the gold standard (9.2% vs. 9.0% and 6.1% vs. 5.4% respectively) (Figure 1). For rehabilitation benefits 1.4% reported to receive it while 1.7% did. For disability pension 17.7%, for rehabilitation benefit 24.1% and for retirement pension 20.6% did not answer.

For disability pension and retirement pension, sensitivity, specificity, negative predicative value (NPV) and agreement for answers only were all above 98% (Table 3). The positive predicative value (PPV) was 93.3% and 83.2% respectively, and Cohen’s kappa was 0.95 and 0.89. When non-responders were set as non-receivers, specificity got higher, PPV remained the same and NPV, sensitivity, Cohen’s kappa and agreement fell. The same trend was found for rehabilitation benefits. Specificity for rehabilitation benefits was higher than the specificity for the two other pensions, while all other measures of validity were lower.

**Table 3:** Validity of self-reported information on disability pension, rehabilitation benefits and retirement pension, the HUNT Study (HUNT2, 1995–1997), N = 40,633 aged 19–87 years

	**Sensitivity (%)**	**Specificity (%)**	**Positive predicative value (%)**	**Negative predicative value (%)**	**Kappa**	**Agreement (%)**
Disability pension						
Answers only	98.7	99.2	93.3	99.8	0.95	99.1
(95% CI)	(98.3–99.1)	(99.1–99.3)	(92.5–94.1)	(99.8–99.9)		
Missing set as non-recipient	95.2	99.3	93.3	99.5	0.94	99.0
(95% CI)	(94.5–95.9)	(99.2–99.4)	(92.5–94.1)	(99.5–99.6)		
Rehabilitation benefits						
Answers only	81.7	99.5	72.2	99.7	0.76	99.2
(95% CI)	(78.3–85.0)	(99.4–99.6)	(68.6–75.9)	(99.6–99.8)		
Missing set at non-recipient	60.4	99.6	72.2	99.3	0.65	98.9
(95% CI)	(56.7–64.0)	(99.5–99.7)	(68.6–75.9)	(99.2–99.4)		
Retirement pension						
Answers only	98.1	98.6	83.2	99.9	0.89	98.6
(95% CI)	(97.5–98.7)	(98.5–98.8)	(81.7–84.7)	(99.8–99.9)		
Missing set as non-recipient	93.9	98.9	83.2	99.7	0.88	98.7
(95% CI)	(92.9–94.9)	(98.8–99.0)	(81.7–84.7)	(99.6–99.7)		

### Characteristics of individuals not responding to questions on benefits, aim two

For disability pension and retirement pension, non-responders were less likely to be receivers than those responding (self-report overestimate rate of reception) (p < 0.001) (Table 4). For rehabilitation benefits, 1.6% of responders and 2.1% of non-responders were receivers (p = 0.004).

**Table 4:** Comparing non-responders and responders, the HUNT Study (HUNT2, 1995–1997), N = 34,262 aged 19–87 years

	**Disability pension**			**Rehabilitation benefit**			**Retirement pension**			
	**Valid answer (N = 28,847)**	**No answer (N = 5,415)**	P-values*	**Valid answer (N = 27,105)**	**No answer (N = 7,157)**	P-values*	**Valid answer (N = 27,853)**	**No Answer (N = 6,409)**	P-values*	**Total (N = 34,262)**
Female	50.9%	57.9%	<0.001	50.4%	57.8%	<0.001	50.3%	59.1%	<0.001	52.0%
Age (mean, in years)	42.4	48.7	<0.001	41.4	51.1	<0.001	42.5	47.4	<0.001	43.4
(SD)	(12.3)	(15.2)		(11.8)	(14.2)		(12.7)			(13.0)
Anxiety	14.7%	18.2%	<0.001	13.9%	20.1%	<0.001	13.6%	22.4%	<0.001	15.2%
Depression	8.6%	11.9%	<0.001	7.9%	13.9%	<0.001	7.9%	14.6%	<0.001	9.1%
Somatic diagnosis >0	11.6%	15.7%	<0.001	10.4%	19.0%	<0.001	11.0%	17.6%	<0.001	12.2%
Physical activity			<0.001			<0.001			<0.001	
*No*	22.3%	23.1%		21.8%	24.8%		21.6%	25.7%		22.4%
*Moderate*	46.3%	51.9%		45.6%	53.0%		46.3%	51.0%		47.2%
*Heavy*	31.5%	25.1%		32.6%	22.2%		32.1%	23.3%		30.5%
Consumption of alcohol			<0.001			<0.001			<0.001	
*No consumption*	25.5%	31.6%		24.1%	35.1%		24.8%	33.3%		26.4%
*Moderate consumption*	71.7%	66.3%		72.9%	62.9%		72.3%	64.4%		70.8%
*Heavy consumption*	2.9%	2.2%		3.0%	2.0%		2.9%	2.3%		2.8%
Smoking	31.4%	37.4%	<0.001	30.9%	37.9%	<0.001	30.7%	39.7%	<0.001	32.4%
Marital status			<0.001			<0.001			<0.001	
*Married*	62.3%	58.7%		61.6%	62.0%		62.4%	58.6%		61.7%
*Not married*	29.0%	24.2%		30.2%	20.7%		29.2%	23.9%		28.2%
*Separated or divorced*	7.3%	8.8%		7.1%	9.3%		6.8%	10.8%		7.6%
*Widow/Widower*	1.5%	8.3%		1.1%	8.0%		1.6%	6.8%		2.6%
Self-reported disability pension	9.1%									7.7%
Receiving disability pension	8.6%	1.5%	<0.001							7.5%
Self-reported rehabilitation benefit				1.8%						1.4%
Receiving rehabilitation benefit				1.6%	2.1%	=0.004				1.7%
Self-reported retirement pension							5.7%			4.6%
Receiving retirement pension							4.6%	0.9%	<0.001	3.9%

*testing if there is a significant difference between those answering and those not answering, chi-square test/T-test.

Non-responders were generally older and more likely to be female. They reported more anxiety, more depression and more somatic diagnosis. They were less physically active and reported a lower consumption of alcohol. They smoked more and were for disability pension and retirement pension less likely to be married.

## Discussion

### Summary of findings

Measures of validity for answers only were very high for both disability pension and retirement pension, somewhat lower for rehabilitation benefits. For all three benefits, sensitivity, specificity and NPV were high, PPV slightly lower. Non-responders were found to be mainly non-receivers. Imputing non-responders as non-receivers led to falling measures of validity - though sensitivity, specificity and NPV were kept above 90% for all benefits except rehabilitation benefits.

### Strengths and limitations

A major strength of this study is that it was conducted in a country with a common public social security system virtually utilized by all. Also, the complete nation-wide registry containing inter alia information on pensions and benefits is a great advantage. The registry data is complete without missing, and the quality of the data is very high. The ability to link this registry, using a unique 11-digit identification number assigned to all individuals living in Norway, to the large, population-based survey, HUNT2, gave ideal conditions for analyzing validity of self-reported data.

In HUNT2 participants were asked “Are you currently receiving any of these public benefits”. The specific date of participation was then linked to the corresponding register-data, reducing the problem of recall bias seen in retrospectively collected data [37].

The study also has some notable limitations. Although the study-sample was large and the participation-rate at baseline was high (69.5%), no data on benefits were available for non-participants. A study investigating HUNT3 (the third wave of the HUNT-study) found the rate of benefits to be higher amongst the non-attendees than amongst participants [38]. This was also the case in HUSK, a comparable study, but with a more limited age-range [39]. Thus, selection bias, possibly reducing generalizability [37], cannot be ruled out.

The pensions here investigated are ususally granted as a 100%, but can also be granted graded. It could have been interesting to investigate differences in validity between individuals receiving benefits as a 100% and those receiving much less. In our study population, however, this was not possible. Amongst individuals receiving disability pension, 0.10% received it as less than 50%. Amongst those receiving rehabilitation benefits, 0.79% received less than 50%. For the major part of individuals receiving retirement pension, we have no data on grading. In a subsample of n = 859 containing this information, 0.02% individuals received less than 50% retirement pension. Due to the small number of individuals receiving less than 50% benefits, we do not consider it ethically justifiable or meaningful to investigate these individuals further.

Also, the data investigated was collected between 1995 and 1997, and the measures of validity found are from this time. Even if the proportion of people granted pensions and benefits might change over time, we see no reason to believe this to affect how truthfully participants report their benefit situation. However, as discussed below, PPV and NPV are measures dependent on the prevalence of the condition studied and might therefore change more with varying prevalence than sensitivity and specificity. Though we do not expect the validity of self-reported pensions and benefits to have changed greatly since 1995, measures of validity should also be investigated in newer datasets.

### Interpreting of findings

The high validity found in this study, and that specificity is higher than sensitivity and NPV higher than PPV, shows that non-receivers can be correctly classified based on self-report. This is in line with previous findings [21].

A reason for lower sensitivity than specificity could be unwillingness to reveal personal circumstances. Reluctance to answer questions, or to answer them correctly, could be expected if people consider the information sensitive. Social desirability has been found important for validity of self-reported information on for instance job performance, citizenship behavior, vitality and dietary intake [8-10]. If this is the case also for self-report of benefit receipt, we would expect to see under-reporting. In our study, however, participants getting their answers wrong mainly over-reported. For disability pension and retirement pension non-responders were less likely to be receivers than the group in total. Social desirability and reluctance to answer sensitive questions correctly can therefore not explain our findings.

False positive answers could be explained if participants knew they were receiving money - but not which benefit - and ticked off for the wrong one. Investigating false positives, for disability pension 43.4% of these, for rehabilitation benefits 22.4% and for retirement pension 20.3% received one of the other benefits evaluated. This percentage might possibly be even higher: Participants here grouped as false positives could receive benefits that we do not have data on (social benefits, survivors’ benefits etc.). This cannot be evaluated in this study.

Another consequence of participants being unsure which benefit they received could be ticking off for more than one benefit. This could also lead to false positive answers. In our data, only n = 45 participants ticked off for two benefits, none for three. This can therefore not explain the over-reporting.

While sensitivity and specificity are independent of the prevalence of the situation being studied, PPV and NPV are not. At low prevalence false positives will tend to overwhelm true positives, resulting in a falling PPV. At the same time, true negative will tend to overwhelm false negative and the NPV will rise with falling prevalence [40]. This is reflected in our study – where, for all three benefits, the prevalence is low and NPV higher than PPV. Further, the prevalence for rehabilitation benefits is lower than the prevalence of the other benefits. This might explain the even lower PPV and the high NPV for this benefit.

Though some participants got their answers wrong, and sensitivity and PPV was somewhat lower than specificity and NPV, estimates of validity were very high. This is in line with previous studies [21,22] and indicates that research based on self-reported data on pensions is of good quality, and that participants answering to a great extent can be trusted.

Problems arise with individuals not answering. For all three benefits investigated, between 17.7% and 24.1% did not answer the specific question on benefits. Studies excluding individuals not answering specific questions might experience a drastic decline of power. Also, systematic differences between responders and non-responders can lead to selection bias when excluding these individuals. In our study, individuals not responding to the questions on benefit receipt reported more anxiety, more depression and more somatic diagnosis. They were less physical activity and smoked more. These findings are to a large extent comparable to characteristics of non-participants and drop-outs [39,41,42].

Research is often focused on individuals with physical, mental or social problems. The fact that these individuals to a larger extent than healthy individuals do not answer, or answer questions incorrectly, should be noted. Excluding non-responders to specific items might therefore exclude the individuals we are interested in investigating.

In order to avoid decline in power and the selection bias seen when excluding non-responders, non-responders could be included in the study. The risk of receiving the benefits we investigated when not responding was 2.1% or less. Our results show that by setting non-responders as non-receivers validity is kept high.

## Conclusion

The validity of self-reported data on disability pension, rehabilitation benefits and retirement pension was found to be high: it seems that participants answering can be trusted. This indicates that self-reported data on different pensions and benefits are useful in epidemiological studies. A considerable proportion of participants, however, do not answer questions on benefits. Excluding these can lead to selection bias and a drastic decline of power. For disability pension and retirement pension non-responders were less likely to be receivers than responders. As 2.1% or less of non-responders were receivers, these participants can be imputed to non-receivers, keeping power and validity high. Due to possible systematic differences between non-participants and participants, future research should seek to examine the possible effect on validity and generalizability caused by selection bias due to non-participation.

## Abbreviations

PPV: Positive predicative value; NPV: Negative predicative value.

## Competing interests

The authors declare that they have no competing interests.

## Authors’ contributions

Mykletun designed the study, while authors Wilhelmsen, Ariansen and Myrtveit contributed further to the scope of the study. Myrtveit conducted literature searches, and provided summaries of previous studies. Myrtveit conducted the statistical analysis under supervision of Ariansen and Mykletun. Krokstad initiated studies on social security benefits in HUNT, provided and quality assured data. All authors contributed to the interpretation of findings, and Myrtveit wrote the first draft of the manuscript. All authors contributed to the further refinement of the manuscript and approved the final version.
